# Psychometric Evaluation of the Patient‐Experienced Continuity of Care Questionnaire (PECQ) Using Rasch Model

**DOI:** 10.1111/hex.70559

**Published:** 2026-02-14

**Authors:** Linda Ljungholm, Mirjam Ekstedt, Cecilia Fagerström, Ingrid Djukanovic, Kristofer Årestedt

**Affiliations:** ^1^ Faculty of Health and Life Sciences Linnaeus University Kalmar Sweden; ^2^ Department of Learning, Informatics, Management and Ethics, Medical Management Centre Karolinska Institutet Stockholm Sweden; ^3^ Department of Research Region Kalmar County Kalmar Sweden

**Keywords:** continuity of care, measurements, nursing, patient experience, patient safety, psychometrics, quality of healthcare, Rasch analysis, reliability, validity

## Abstract

**Background:**

The Patient‐Experienced Continuity of care Questionnaire (PECQ) was developed to measure continuity of care (CoC) in primary care, but has only been evaluated in accordance with classical test theory, which has some weaknesses compared with modern test theory.

**Aim:**

The aim of this study was to evaluate the measurement properties of the PECQ using the Rasch model.

**Method:**

Data for this psychometric study were collected using questionnaires administered to patients aged 18 years and older with one or more chronic conditions. Participants were consecutively recruited from 18 public primary care centres. The final sample comprised 324 patients (54% female and 46% male) with a mean age of 71.8 years (SD = 12).

**Results:**

The PECQ demonstrated satisfactory measurement properties in terms of individual item fit, response category functioning, targeting and differential item functioning by age. Minor issues were identified regarding global model fit, local independence, reliability and non‐uniform differential item functioning by sex.

**Conclusions:**

Overall, the results show that PECQ has sound psychometric properties in adult patients with chronic disease and can be used to measure CoC in primary care settings.

**Impact:**

This study addresses the need for accurate measurement of patient experiences of CoC, and the Rasch model was therefore used to evaluate the PECQ. As the instrument demonstrated good fit to the Rasch model, the findings have important implications for healthcare researchers, policymakers and practitioners. The PECQ provides a scientifically sound tool for evaluating CoC, enabling more accurate quality assessments and supporting targeted interventions in both Swedish and international primary care contexts where the instrument is adapted.

**Patient or Public Contribution:**

Patients were actively involved in the study by participating in the cognitive validation of the questionnaire items. Their contributions helped ensure that the content was understandable, relevant and appropriately tailored to the target population. By providing feedback on the wording and content, the patients contributed to the development of a user‐friendly and contextually grounded instrument, which was subsequently validated in this study using the Rasch model.

**Abbreviations**
CoC: continuity of care
CTT: classical test theory
PECQ: The Patient‐Experienced Continuity of care Questionnaire

## 1 Background

A growing ageing population with an increasing need for coordinated healthcare services is one of the biggest challenges for the sustainability of healthcare systems around the world [1, 2]. Fragmented care places high demands on continuity of care (CoC), particularly for patients with multiple chronic conditions. As more people with complex care needs are being cared for in their own homes, primary care has increased in importance [3]. Primary care has a coordinating role as the patients’ first point of contact with care and plays a central role in ensuring continuity and integration [4]. CoC is claimed to be an indicator of high quality in primary care [5] and an important characteristic of safe and efficient care, where relational, informational and management continuity contribute to patient safety and trust [6, 7].

To meet this need, the Patient‐Experienced Continuity of care Questionnaire (PECQ)—a patient‐reported experience measure covering four dimensions of continuity (Information, Relation, Management and Knowledge) for patients with complex care needs in a primary care setting—has been developed [8]. In the development of the PECQ, content validity was supported based on the assessment of the content validity index among experts and cognitive interviews by patients with complex care needs. In addition, the instrument was psychometrically evaluated in the context of classical test theory (CTT). The hypothesized four‐factor structure was confirmed through CFA with satisfactory model fit (CFI, TLI, SRMR, RMSEA). Factor loadings were high (0.74–0.88), indicating that items contributed well to their respective constructs. Scale correlations were moderate to high (raw scores: 0.69–0.81), suggesting related but distinct dimensions. Internal consistency was strong (ordinal alpha: 0.87–0.91; omega: 0.81–0.89), and test–retest reliability was good (ICC: 0.86–0.88), demonstrating stability over time [8]. However, CTT has some significant weaknesses. For example, it is sample‐ and test‐dependent [9], and the reliability and standard error of measurement are assumed to be constant for all test takers [10]. These problems are addressed in the Rasch model, which can be used to evaluate important psychometric aspects such as unidimensionality, local independence, response category functioning, targeting, reliability and differential item functioning (DIF) [10]. Therefore, this study aimed to evaluate the measurement properties of the PECQ using the Rasch model.

## 2 Methods

### 2.1 Design

This psychometric study was based on data from the study in which PECQ was developed. These data were collected between August 2021 and May 2022 [8].

### 2.2 Participants and Procedure

Data were collected in 18 of 39 public primary care centres in a region of southeast Sweden. Participant inclusion criteria were being 18 years and older, having at least one chronic disease and having one or more care contacts. Patients who fulfilled the inclusion criteria were consecutively informed by physicians and/or nurses at the care units (*n* = 657). Patients who were interested in participating (*n* = 603) received a questionnaire to complete at home, along with a stamped return envelope. In total, 326 patients accepted participation by completing and returning the questionnaire (response rate 54%). Two patients had not completed any item in the PECQ and were excluded from the present study, giving a final sample of 324 participants.

### 2.3 The Questionnaire

The questionnaire included demographic and medical data such as age, sex, chronic disease and number of care contacts. It also contained the PECQ, which consists of 20 items covering four dimensions (i.e., scales): Information (four items), Relation (six items), Management (five items) and Knowledge (five items). For each item, a response is given on a four‐point Likert scale, ranging from 1 ‘Strongly disagree’ to 4 ‘Strongly agree.’ Each scale is scored by summarizing the item responses and dividing the sum by the number of items in the scale. Thus, all scales have a possible score range between 1 and 4; higher scores reflect higher levels of CoC.

### 2.4 Statistical Analysis

Descriptive statistics were used to present the characteristics of the participants and study variables, mean and standard deviations for continuous variables and frequencies for categorical variables. These analyses were conducted in R for Windows, version 4.2.2 (The R Foundation for Statistical Computing, Vienna, Austria), including the package summary tools 1.0.1.

The PECQ was evaluated based on the Rasch model using the RUMM2030 for Windows, version 5.8.1 (Rumm Laboratory Pty Ltd, Duncraig, Australia). As the items in the PECQ have a Likert response format, the polytomous Rasch model was applied (partial credit model). The analysis was based on six class intervals to ensure a sufficiently large number of persons in each class (*n* ≥ 30). Although some data were missing (between 2% and 5% across items), all cases with at least one valid response were retained for analysis.

Model fit was evaluated regarding global fit and individual item fit. To support global fit, the mean of the standardized item fit residual should be close to 0, and the corresponding standard deviation close to 1. In addition, the total item trait interaction statistics (*χ* ^2^‐based statistics) should be non‐significant. To support individual item fit, standardized item fit residuals should fall within the range of −2.5 to 2.5, and Bonferroni corrected probability values, calculated as the significance level divided by the number of items, should be non‐significant (*p* < 0.013 for the Information scale, *p* < 0.008 for the Relation scale and *p* < 0.010 for the Management and Knowledge scales). Item characteristic curves were also used to graphically examine individual item fit. To support person fit, the mean of the standardized person fit residual should be close to 0 and the corresponding standard deviation close to 1, and the distribution of standardized person fit residuals should be within the range of −2.5 and 2.5 [11, 12].

To evaluate the response category function, the ordering of the item threshold order was examined. Disordered thresholds indicate that the response categories do not function as intended, that is, from less to more [11, 12].

A principal component analysis of item residuals was used to evaluate unidimensionality [13, 14]. Items with the strongest positive versus negative factor loadings on the first component were considered to represent potentially different dimensions and formed two new item subsets. The item subsets were then used to calculate new person measures and associated standard errors for each participant. Then, dependent sample *t*‐tests were conducted to compare person measures from the two item subsets. To support unidimensionality, less than 5% of the *t*‐tests are expected to be significant (*p* < 0.05).

Correlations between item residuals were examined to evaluate local independence, that is, if each item provides related, but independent information. Local independence is reflected by low correlations between item residuals; correlations greater than 0.2 above the average of all residual correlations, referred to as relative Q3, are suggested to be problematic [15].

To evaluate targeting, the person‐item threshold distribution was examined. To support targeting, the item threshold logits should cover about the same range as person location logits [11].

Reliability was evaluated with the person separation index and ordinal alpha. Coefficients larger than 0.7 are expected to support reliability for both measures [12, 16]. To facilitate comparisons with other studies, Cronbach's alpha was also reported.

To detect DIF for age (< 65 years vs. ≥ 65 years) and sex (females vs. males), a two‐way analysis of variance (fixed‐effects model) was used, as implemented in RUMM2030. The dependent variable was the residuals from the Rasch model, while the independent variables were group (age and sex) and class interval (ability level) [12]. Both the main effect of group and interaction effect of group and class intervals were used to detect DIF; the first reflects uniform DIF and the later non‐uniform DIF. These analyses were Bonferroni‐corrected, that is, *p* < 0.004 was used for the Information scale and *p* < 0.003 for the Relation scale, Management scale and Knowledge scale.

## 3 Results

### 3.1 Participant Characteristics

The sample included 324 participants, 54% female and 46% male, with a mean age of 71.8 years (SD = 12.2). Additionally, 67.2% of the males and 32.8% of the females were living alone. In total, 51.2% participants had more than one self‐reported disease, and the median number of care contacts was 2 (q1–q3 = 1–3, range = 1–8). The most common self‐reported diagnosis was cardiovascular disease (*n* = 112), followed by diabetes (*n* = 107), lung disease (*n* = 51), cancer (*n* = 44), rheumatic disease (*n* = 42), psychiatric disease (*n* = 32) and stroke (*n* = 5). Other diseases were reported by 156 participants.

### 3.2 Measurement Properties of PECQ

#### 3.2.1 Model Fit

Regarding global fit (Table 1), the mean of standardized item fit residual values was all close to 0 in the scales: Information (−0.12), Relation (−0.10), Management (0.10) and Knowledge (0.20). The corresponding standard deviation was close to 1 for the Information scale (1.15), Management scale (0.97) and Knowledge scale (0.91), but larger for the Relation scale (1.66). Further, the total item trait interaction statistics supported the hypothesized measurement model for the Management scale (*p* = 0.863) and Knowledge scale (*p* = 0.951), but not for the Information (*p* < 0.001) and Relation (*p* = 0.047) scales.

**Table 1:** Global fit statistics for items, scale score distribution and reliability for the four scales of the Patient‐Experienced Continuity of care Questionnaire.

	Information scale	Relation scale	Management scale	Knowledge scale
Total item trait interaction^a^				
Total item *χ* ^2^	47.1	44.5	17.5	14.5
df	20	30	25	25
*p*‐value	< 0.001	0.047	0.863	0.951
Standardized item fit residual				
Mean (SD)	−0.12 (1.15)	−0.10 (1.66)	0.10 (0.97)	0.20 (0.91)
Standardized person fit residual				
Mean (SD)	−0.41 (0.95)	−0.34 (1.08	−0.40 (1.11)	−0.36 (1.09)
Scale score distribution^b^				
Mean (SD)	3.22 (0.58)	2.80 (0.70)	2.93 (0.72)	2.76 (0.66)
Median (IQR)	3.25 (3˗3.75)	2.83 (2.3˗3.3)	3.0 (2.4˗3.6)	2.80 (2.4˗3.2)
Min–Max	1˗4	1˗4	1˗4	1˗4
Reliability metrics				
Person separation index	0.70	0.83	0.80	0.80
Ordinal alpha	0.87	0.91	0.92	0.88
Cronbach's alpha	0.79	0.85	0.87	0.82

*Note:* The Information scale includes Items 1–4, the Relation scale includes Items 5–10, the Management scale includes Items 11–15 and the Knowledge scale includes Items 16–20.

^a^ The *χ* ^2^ statistic reflects the deviation between observed and expected item scores across class intervals, while the degrees of freedom (df) equal the number of items multiplied by the number of class intervals minus one. The level of statistical significance is *p* < 0.05.

^b^ Raw scores for each scale have a possible range between 1 and 4.

**Figure 1:**
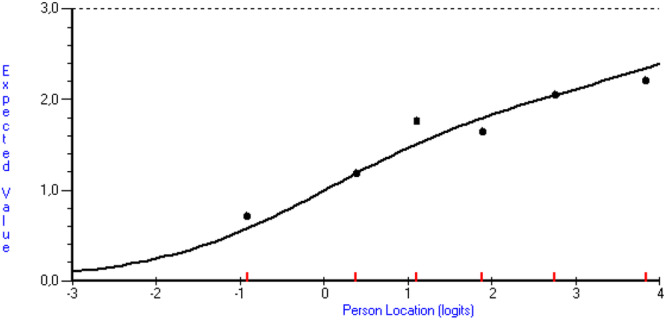
Item characteristic curve for Item 4 in the Information scale. The solid line represents the expected probability of a correct response across the latent trait continuum (ability). The dots indicate observed class intervals, which should align closely with the expected curve if the item fits the model well.

Regarding individual item fit (Table 2), standardized item fit residuals were within the range of ±2.5 for all items. Only Item 4 in the Information scale (healthcare professionals having information about the patient's past) deviated significantly from the Rasch model (*p* = 0.006). However, the standardized item fit residual was acceptable (1.51), and the item characteristic curve did not indicate any large deviation between model and data (Figure 1).

**Table 2:** Item location, individual item fit statistics and item thresholds for the four scales of the Patient‐Experienced Continuity of care Questionnaire.

Items	Item location	Individual item fit statistics		Item thresholds		
		Standardized item fit residual	*p‐*value^a^	I	II	III
Information scale						
1. I get comprehensible information from healthcare to understand my diseases and treatments	−0.33	−0.14	0.066	−2.57	−1.34	2.91
2. I get information on how return visits or follow‐ups will take place	−0.63	−0.81	0.115	−2.80	−0.90	1.80
3. I get comprehensible information on the results of my tests and examinations (e.g., X‐ray)	−0.49	−1.03	0.039	−2.80	−0.63	1.97
4. When I meet new healthcare personnel, they are always informed about what other healthcare personnel have done previously	1.45	1.51	**0.006**	−0.74	0.79	4.29
Relation scale						
5. I feel safe with the care I get, regardless of which healthcare personnel is taking care of me	−1.39	0.88	0.609	−4.25	−1.45	1.54
6. I always meet the same healthcare personnel at each care visit	1.08	−1.05	0.634	−0.19	0.60	2.83
7. All healthcare personnel take me seriously	−1.17	0.31	0.336	−2.82	−1.74	1.06
8. I always know who is responsible for my care	0.54	−2.11	0.019	−0.68	0.44	1.84
9. Healthcare personnel always ask about other diseases in addition to the one I sought care for	1.33	2.50	0.040	−0.50	1.35	3.14
10. I always know who I can turn to with questions about my care and treatments	−0.39	−1.11	0.250	−2.16	−0.33	1.32
Management scale						
11. Healthcare personnel have always reviewed my disease history ahead of the visit	0.02	−0.44	0.831	−2.10	−0.52	2.69
12. Healthcare personnel regularly follow up on my diseases and treatments	0.25	0.72	0.516	−1.52	−0.28	2.54
13. Healthcare personnel always help me coordinate my healthcare visits	0.76	0.70	0.573	−0.80	0.38	2.69
14. Care and treatments are available to me when I need them	−0.92	0.86	0.883	−3.15	−1.42	1.80
15. Everyone works towards clearly set goals regarding my care	−0.10	−1.36	0.352	−1.79	−0.68	2.16
Knowledge scale						
16. I always participate in the planning of my care	−0.25	0.58	0.907	−1.95	−0.75	1.95
17. I always know what I can do myself to manage my diseases and treatments	−0.71	0.85	0.914	−2.84	−1.12	1.84
18. Healthcare personnel always ask me what I feel is significant in my everyday life	1.18	0.78	0.602	−0.86	1.10	3.31
19. Healthcare personnel always ask questions to ensure that I have understood the information correctly	0.11	−1.34	0.401	−1.94	−0.11	2.38
20. I can always find comprehensible information about my care	−0.34	0.13	0.742	−2.60	−0.70	2.27

^a^ Bonferroni‐corrected significance levels: *p* < 0.013 for the Information scale, *p* < 0.008 for the Relation scale and *p* < 0.010 for the Management and Knowledge scales. Significant *p*‐values that indicate misfit are marked with bold.

Regarding person fit, the mean of standardized person fit residual values were all below 0 in the scales, while the corresponding standard error was close to 1: Information (*M* = −0.41, SD = 0.95), Relation (*M* = −0.34, SD = 1.08), Management (*M* = −0.40, SD = 1.11) and Knowledge (*M* = −0.36, SD = 1.09). The number of persons with standardized person fit residuals outside the expected range of ±2.5 was largest for the Management scale (*n* = 23), followed by the Information (*n* = 17), Knowledge (*n* = 15) and Relation (*n* = 10) scales.

#### 3.2.2 Response Category Function

No disordered thresholds were identified for any of the four scales, which indicates that the response categories worked as intended (Table 2).

#### 3.2.3 Unidimensionality

Based on the principal component analysis of item residuals, two subsets of items were examined for each scale. The subsets included the following item pairs: 1 and 3 versus 2 and 4 in the Information scale, 6 and 8 versus 7 and 9 in the Relation scale, 12 and 13 versus 14 and 15 in the Management scale and 16 and 17 versus 18 and 19 in the Knowledge scale. Based on the dependent sample *t*‐test of the person measures from the two subsets, fewer than 5% of the participants had significantly different scores in any of the scales: Information 2.2%, Relation 3.4%, Management 3.7% and Knowledge 4.4%. Therefore, the assumption of unidimensionality was supported.

#### 3.2.4 Local Independence

Problems with local dependency were shown in three of the four scales; between Items 5 and 7 (‘I feel safe with the care I get, regardless of which healthcare personnel is taking care of me’ vs. ‘All healthcare personnel take me seriously’) in the Relation scale (*r* = 0.037, Q3 = 0.011), between item 14 and 15 (‘Care and treatments are available to me when I need them’ vs. ‘Everyone works towards clearly set goals regarding my care’) in the Management scale (*r* = −0.008, Q3 = −0.046), and between Items 18 and 19 (‘Healthcare personnel always ask me what I feel is significant in my everyday life’ vs. ‘Healthcare personnel always ask questions to ensure that I have understood the information correctly’) in the Knowledge scale (*r* = 0.052, Q3 = −0.047).

#### 3.2.5 Targeting

**Figure 2:**
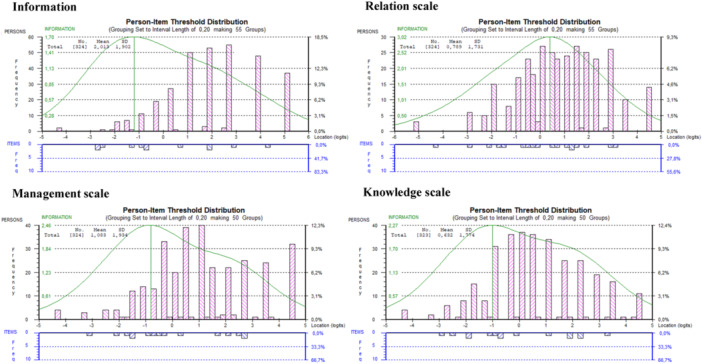
Distribution of person ability measures and item thresholds for the four scales of the Patient‐Experienced Continuity of care Questionnaire (PECQ). Higher logit scores indicate persons with greater ability and items of greater difficulty. The curve represents the test information function, which indicates how much measurement precision the scale provides across different levels of person ability. Higher points on the curve reflect greater precision at those ability levels.

The person‐item threshold distribution showed acceptable targeting for the four scales. The item thresholds covered at least ±3 logits of the person ability, that is, location. However, none of the scales covered persons with very low or very high ability levels, that is, experience of CoC (Figure 2).

#### 3.2.6 Reliability

All scales showed acceptable reliability based on the person separation index: Information 0.70, Relation 0.81, Management 0.78 and Knowledge 0.78. Also, ordinal alpha values were all above 0.7 for the scales: Information 0.87, Relation 0.91, Management 0.92 and Knowledge 0.88 (Table 1).

#### 3.2.7 DIF

**Figure 3:**
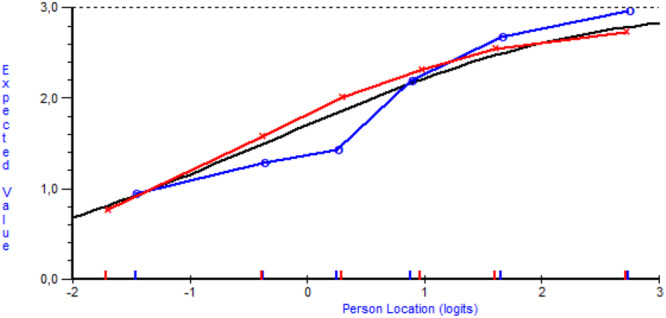
Item characteristic curve for Item 10 with class intervals split by sex to examine differential item functioning (DIF). The solid line represents the expected probability of a correct response based on the Rasch model. Dots indicate observed values for men, and crosses indicate observed values for females. Deviations between the two groups suggest potential DIF; in this case, males tend to score lower than females at lower ability levels, while the opposite pattern appears at higher ability levels.

Neither uniform nor non‐uniform DIF was detected for age. For sex, no uniform DIF was detected, but Item 10 in the Relation scale demonstrated non‐uniform DIF according to the interaction term between sex and class interval in the two‐way ANOVA (*p* = 0.002) (Table 3). An inspection of the item characteristic curve for Item 10, with class intervals split for sex, showed that males tend to score lower than females at lower ability levels on the Relation scale, while the opposite pattern appears at higher ability levels (Figure 3).

**Table 3:** Tests of uniform and non‐uniform differential item functioning (DIF) for age and sex using two‐way ANOVA.

Scales and items	DIF for age^a^		DIF for sex^a^	
	Main effect of age	Interaction effect of age and class intervals	Main effect of sex	Interaction effect of sex and class intervals
Information scale				
1	0.509	0.265	0.762	0.956
2	0.380	0.020	0.263	0.276
3	0.899	0.797	0.180	0.067
4	0.290	0.056	0.624	0.599
Relation scale				
5	0.310	0.344	0.005	0.944
6	0.997	0.650	0.045	0.228
7	0.366	0.337	0.900	0.420
8	0.548	0.497	0.835	0.313
9	0.734	0.143	0.329	0.703
10	0.533	0.185	0.686	**0.002**
Management scale				
11	0.457	0.160	0.421	0.058
12	0.375	0.125	0.799	0.038
13	0.120	0.098	0.566	0.438
14	0.244	0.320	0.970	0.312
15	0.557	0.877	0.137	0.218
Knowledge scale				
16	0.738	0.250	0.631	0.971
17	0.455	0.411	0.991	0.387
18	0.275	0.223	0.482	0.315
19	0.826	0.420	0.438	0.533
20	0.280	0.033	0.840	0.238

*Note:* Main effects represent uniform DIF and interaction effects non‐uniform DIF. Bold *p*‐values indicate DIF.

^a^ Bonferroni‐corrected significance levels: *p* < 0.004 for the Information scale and *p* < 0.003 for the Relation, Management and Knowledge scales. Significant *p*‐values that indicate DIF are marked with bold.

## 4 Discussion

This study aimed to evaluate PECQ using the Rasch model. Overall, the results showed that all four scales demonstrated good measurement properties regarding response category functioning, unidimensionality, local independence, targeting and reliability. In addition, no DIF for age was present, whereas only one item in the Relation scale demonstrated DIF for sex.

Global fit was supported for the Management and Knowledge scales, whereas the Information and Relation scales deviated significantly from the Rasch model. Global fit is affected by sample size and should be interpreted with caution [12]. This is especially true for the Relation scale, which demonstrated a *p*‐value close to 0.05. Therefore, it is also important to evaluate individual item fit. In these analyses, all items demonstrated good model fit based on the standardized item fit residuals. Though Item 4 in the Information scale demonstrated a significant deviation from the Rasch model, the standardized item fit residual was acceptable, and it showed acceptable fit based on the item characteristic curve. One possible explanation for this result is that patients may find it difficult to answer the question, as they are often unaware of whether healthcare professionals are consistently informed about the actions of their colleagues. Furthermore, the phrasing and syntactic structure of the item are more complex compared to the other items. However, the item was retained in the scale due to its theoretical relevance, as it addresses an important aspect of CoC (i.e., whether healthcare personnel have information about what other healthcare personnel have done in the past). To conclude, these analyses support model fit for all scales in the PECQ.

The principal component analysis of item residuals and the *t*‐test approach [12] showed no problems with unidimensionality for any of the scales in the PECQ. Both CTT and Rasch analysis support the underlying structure of PECQ. CFA confirmed the hypothesized four‐factor model (*Information, Relation, Management* and *Knowledge*), demonstrating satisfactory fit and strong factor loadings, which indicate construct validity [8]. Unidimensionality is a key assumption of both CTT and item response theory, like the Rasch model. It implies that the items reflect the differences in the latent construct and that each item assesses a unique aspect of the trait [12]. This convergence strengthens evidence that PECQ comprises four distinct yet related dimensions of CoC.

Some violations of local independence were detected in all scales except the Information scale. Like unidimensionality, local independence is also a key assumption of both CTT and item response theory, such as the Rasch model, and implies that there should not be any correlation between the residuals of two items, that is, the residual correlation should be close to zero after the effect of the latent variable is conditioned out [12, 17]. The violations of local independence were only related to one item pair in each of the three scales and are probably also of minor importance, as the residual correlations were close to the relative Q3 for each scale and did not exceed 0.2 [18]. In addition, no residual correlation was close to traditional recommendations of 0.2 or 0.3 [15]. Problems with local independence can be related to problems with unidimensionality and/or response dependency [12]. Whereas no problems with unidimensionality were shown in the present study, these findings may indicate problems with response dependency, that is, that the answers are influenced by the person's answer to previous items—this is common when test items are similarly worded [17]. During the development of the PECQ, items that were perceived as similarly worded were removed.

The present study did not detect any issues with uniform or non‐uniform DIF related to age. However, one item on the Relational scale—‘I always know who I can turn to with questions about my care and treatments’—exhibited non‐uniform DIF with respect to sex. The graphical examination of the item characteristic curve split for sex showed that females followed the curve as expected, while males showed a steeper curve than anticipated by the model. This interaction effect, manifested as non‐uniform DIF, suggests that the item may be over‐discriminating in males but not in females. The underlying reason for this pattern remains unclear, and it cannot be ruled out that the finding is an artefact. Until further evidence is available, gender‐based comparisons using the Relational scale should therefore be interpreted with caution. However, the Information, Management and Knowledge scales can be used to make invariant measurements between groups of different ages and sexes. The present study also demonstrated satisfactory reliability for all the PECQ scales, good targeting and excellent response category functioning. CTT results corroborate these findings, showing strong internal consistency (ordinal alpha: 0.87–0.91) and good test–retest reliability (ICC: 0.86–0.88) [8], while the present Rasch analysis supported reliability through person separation indices ≥ 0.70.

## 5 Limitations

This study has some limitations that should be considered. The Rasch converts ordinal data into interval‐level measures [12] and was therefore applied to provide a more rigorous evaluation of PECQ's measurement properties compared to CTT, which has previously been used to evaluate the instrument [8]. The Rasch model is prescriptive, requiring data to fit the model perfectly to produce invariant measures. Alternative IRT models allow greater flexibility by estimating discrimination and guessing parameters. This implies that these models are more exploratory, aiming to find the best fit for the data. In addition, they require larger samples and are more complex [10]. While Rasch provides robust and interpretable results, its strict assumptions may be a limitation [12, 19].

Selection bias cannot be excluded, as the study design did not permit a drop‐out analysis. However, the sample demonstrated variation in age, sex, diagnosis and education, and it meets the recommended sample size guideline of 10–20 participants per threshold for the polytomous Rasch model [12]. For the PECQ, the number of thresholds is 12 for the Information scale, 15 for the Management and Knowledge scales and 18 for the Relation scale. Consequently, the required sample sizes for these scales should be at least 120, 150 and 180 participants, respectively.

Missing data in Rasch analysis can affect item parameter estimation and model fit, particularly when the missingness mechanism is not completely random [20, 21, 22]. Previous studies suggest that excluding cases may introduce bias and compromise validity [22]. In this study, all available valid responses were included, following the standard procedure implemented in the RUMM 2030 software.

Another limitation is that the study was based on the same sample as we used in the development of the PECQ [8]. Though the Rasch model theoretically is expected to be sample‐independent, in contrast to CTT, the PECQ may need to be evaluated in a new sample.

Finally, in the present study, DIF was evaluated for age and sex. In future studies, DIF should be assessed for other groups, such as comorbidity, care consumption and living situation.

## 6 Conclusion

This study contributes important knowledge about the measurement properties of PECQ from the perspective of the Rasch model. The results suggest that the instrument has sound and robust psychometric properties among adult patients in primary care, with only minor issues related to local independence and DIF for sex, which are unlikely to substantially affect validity but warrant further exploration in future studies. As a patient‐reported experience measure, PECQ can complement traditional CoC indices (e.g., designated physician or care contact) in clinical quality improvement and research, enabling more accurate assessments of care quality.

## Author Contributions

**Linda Ljungholm:** conceptualization, methodology, data curation, formal analysis, writing – original draft preparation, writing – review and editing, visualization. **Kristofer Årestedt:** statistician consulting, methodology, formal analysis, writing – original draft preparation, writing – review and editing, visualization. **Cecilia Fagerström:** writing – review and editing. **Ingrid Djukanovic:** writing – review and editing. **Mirjam Ekstedt:** conceptualization, methodology, writing – original draft preparation, writing – review and editing, funding acquisition and project administration.

## Ethics Statement

The study was approved by the Swedish Ethical Review Authority [Dnr 2018/23‐31, approved 20180321 and 2021‐01320, approved 20210502], Department of Health and Nursing Science.

## Consent

Participation in the study was voluntary, and participants could withdraw from the study at any time. The participants received verbal and written information about the study, and consent was given by submitting the questionnaire. The researchers have observed the rules that apply to research with people under the Helsinki Declaration [23].

## Conflicts of Interest

The authors declare no conflicts of interest.

## Data Availability

The datasets generated and analysed in the current study are not publicly available, to protect the privacy of the participants, as per the Swedish Ethics Review Authority's guidelines. The copyright belongs to the authors.
